# Herpes Simplex Virus Type 2 Mucin-Like Glycoprotein mgG Promotes Virus Release from the Surface of Infected Cells

**DOI:** 10.3390/v13050887

**Published:** 2021-05-12

**Authors:** Edward Trybala, Nadia Peerboom, Beata Adamiak, Malgorzata Krzyzowska, Jan-Åke Liljeqvist, Marta Bally, Tomas Bergström

**Affiliations:** 1 Department of Infectious Diseases, Section for Clinical Virology, Institute of Biomedicine, University of Gothenburg, SE-413 46 Göteborg, Sweden; edward.trybala@microbio.gu.se (E.T.); beatada@yahoo.co.uk (B.A.); krzyzowskam@yahoo.com (M.K.); jan-ake.liljeqvist@microbio.gu.se (J.-Å.L.); 2 Department of Physics, Chalmers University of Technology, SE-412 96 Göteborg, Sweden; nadiapeerboom@gmail.com; 3 Department of Clinical Microbiology, Umeå University, SE-901 85 Umeå, Sweden; marta.bally@umu.se; 4 Wallenberg Centre for Molecular Medicine, Umeå University, SE-901 85 Umeå, Sweden

**Keywords:** herpes simplex virus type 2, glycoprotein mgG, mucin-like protein, virus release, glycosaminoglycans, single particle analysis

## Abstract

The contribution of virus components to liberation of herpes simplex virus type 2 (HSV-2) progeny virions from the surface of infected cells is poorly understood. We report that the HSV-2 mutant deficient in the expression of a mucin-like membrane-associated glycoprotein G (mgG) exhibited defect in the release of progeny virions from infected cells manifested by ~2 orders of magnitude decreased amount of infectious virus in a culture medium as compared to native HSV-2. Electron microscopy revealed that the mgG deficient virions were produced in infected cells and present at the cell surface. These virions could be forcibly liberated to a nearly native HSV-2 level by the treatment of cells with glycosaminoglycan (GAG)-mimicking oligosaccharides. Comparative assessment of the interaction of mutant and native virions with surface-immobilized chondroitin sulfate GAG chains revealed that while the mutant virions associated with GAGs ~fourfold more extensively, the lateral mobility of bound virions was much poorer than that of native virions. These data indicate that the mgG of HSV-2 balances the virus interaction with GAG chains, a feature critical to prevent trapping of the progeny virions at the surface of infected cells.

## 1. Introduction

The initial interaction of many different viruses with cells occurs via binding to ubiquitous cell surface carbohydrate components such as sialic acid residues or glycosaminoglycan (GAG) chains of proteoglycans. However, the same sugar receptor residues that facilitate the virus binding to cells may serve as a trap that precludes the release of progeny virions from the surface of infected cells. Some viruses that bind to sialic acid for example, influenza viruses encode the receptor-destroying enzyme sialidase [1,2], and studies using both a sialidase inhibitor [3] and virus mutants defective in sialidase activity [4] demonstrated the presence of large clumps of progeny virus particles trapped at the cell surface and cross-linked via sialic acid of their own envelope glycoproteins. Because the virus release from infected cells was vulnerable to antiviral intervention [3] it is not surprising that the drugs currently approved for the treatment of influenza virus infections are inhibitors of sialidase [5].

A number of viruses, including herpes simplex virus (HSV) [6], target GAG chains of heparan sulfate (HS) and chondroitin sulfate (CS) for efficient attachment of virus particles to cells. HSV-1 binding to HS requires a dodecasaccharide fragment of an HS chain comprising a minimum of one 2-*O*- and one 6-*O*-sulfate group at specific spacing [7], while the binding of this virus to CS chains was greatly promoted by the presence of CS-E units [8]. In contrast to extensive studies reported on the virus attachment to cell surface GAG chains (reviewed in [9]), little is known of how the virus-GAG interaction is balanced at the stage of the release of progeny virions from the surface of infected cells. In this respect, it is worth noting that the synthesis of many cellular proteins, including those that could trap viral particles at the cell surface, such as tetherin [10,11], is efficiently shut off during the course of HSV infection of cells. However, the expression of the GAG-bearing proteoglycans, such as some syndecans, was found to be either up- [12] or down-regulated [13] by different herpesviruses. Although a number of different viruses can interact with GAG chains, no GAG-degrading enzymatic activity encoded by a virus has so far been reported. Instead, Hadigal et al. [14] found that the expression of cellular heparanase, an HS-degrading enzyme, was enhanced in cells infected with HSV, and that the knockdown of the heparanase gene decreased release of HSV-1 particles from the surface of cultured cells by ~2–4 fold. We have previously found that the GAG mimetics muparfostat (formerly known as PI-88) and heparin targeted the glycoprotein components of HSV that comprise numerous and clustered *O*-linked glycans, known as mucin-like regions. In particular, HSV-1 variants resistant to GAG mimetics expressed gC lacking an entire amino-terminal mucin-like region [15] while HSV-2 variants were deficient of gG [16], a glycoprotein derived from proteolytic cleavage of a precursor into a mature cell- and virion membrane-associated fragment (mgG) that comprises mucin-like structures, and a secreted fragment (sgG) [17,18,19,20]. These observations indicate that the mucin-like domains of gC of HSV-1 and mgG of HSV-2 play a key role in determining the specific sensitivity of respective virus to GAG mimetics and are most likely involved in the regulation of the virus-GAG interplay at different stages of the HSV life cycle. The sgG was found to potentiate the chemokine function [21] through the stabilization of chemokine receptor [22].

We have previously found that the mgG-deficient mutant of HSV-2 produced little amounts of infectious virus in the supernatant medium of infected cells [16]. In the present work, we investigated whether mgG of HSV-2 could promote the release of virus particles from infected cells. Our study demonstrated that (i) the mgG deficient HSV-2 virions were present on the surface of infected cells but were poorly released into the supernatant medium; (ii) the GAGs or their mimetics such as muparfostat or heparin could liberate these virus particles almost to the native HSV-2 level, and that (iii) the mgG deficient mutant virus exhibited increased binding to GAG chains, but poorer lateral mobility of bound virions in comparison to native HSV-2, suggesting that this interaction was greatly unbalanced and shifted towards the trapping of viral particles on the GAG chains.

## 2. Materials and Methods

### 2.1. Materials

The muparfostat (previously denoted as PI-88) was provided by Progen Pharmaceuticals Ltd., Brisbane, Australia and Medigen Biotechology Corporation, Taipei, Taiwan. Heparin was obtained from Medicarb (Stockholm, Sweden). Methyl-^3^H-thymidine (20 µCi/mL; specific activity 6.7 Ci/mmole) and Na_2_^35^SO_3_ (Sulfur-35 radionuclide, specific activity 1050–1600 Ci/mmole) were purchased from Perkin Elmer. Mucin type I-S from bovine submaxillary glands was obtained from Sigma-Aldrich (St. Louis, MO, USA) (M3895). Monoclonal antibody 4A5A9 against glycoprotein sgG of HSV-2, which recognizes linear epitope 206-APPQA-210 [23] and monoclonal antibody O1C5 (anti-glycoprotein mgG of HSV-2), whose reactivity was mapped to linear epitope 557-HRGGPEE-563 [24], were prepared as described previously [23,24]. Monoclonal antibodies B11D8 (anti-glycoprotein gB of HSV) and B1E6 (anti-glycoprotein gE of HSV) were identified as specific for respective proteins by radioimmunoprecipitation and/or western blot as described previously [25]. Monoclonal antibody E5F7 that recognizes glycoprotein gC of HSV-2 in immunoaffinity chromatography [16] was also used. Stocks of anti-gB, anti-gC, anti-gE and anti-gG monoclonal antibodies were prepared by growing hybridoma cells in dialysis tubing [26]. Rat polyclonal anti-Us3 protein of HSV was raised against baculovirus-expressed GST-Us3 fusion protein and its specificity was confirmed by western blot [27]. This antiserum was kindly provided by Dr. B. Banfield, Department of Microbiology and Immunology, Queen’s University, Kingston, Canada.

### 2.2. Cells and Viruses

African green monkey kidney (GMK AH1) cells [28], obtained from the Swedish Institute for Infectious Disease Control, Stockholm, were cultivated in Eagle’s minimum essential medium (EMEM) supplemented with 2% fetal calf serum (FCS), 0.05% Primaton RL substance (Kraft Inc., Norwich, CT, USA), 60 µg/mL of penicillin and 100 µg/mL of streptomycin. Vero, human laryngeal epidermoid carcinoma (HEp-2), human skin keratinocytes (HaCaT) [29], and murine L and mutant sog9 [30] cells were propagated in Dulbecco’s modified EMEM supplemented with 10% fetal calf serum, 1% l-glutamine, 1% tricine (Vero) and antibiotics. HEp-2 cells were obtained from the Swedish Institute for Infectious Diseases Control, Stockholm. HaCaT cells were obtained from Dr. N.E. Fusenig, German Cancer Research Center, Heidelberg. Murine L and sog9 cells were obtained from Dr. F. Tufaro, University of British Columbia, Vancouver. All cell lines used were free from mycoplasma contamination. The HSV-2 strain 333 (wt333) was used [31]. The mgG deficient mutant viruses designated 333 + AC9gG (mgG-def) and 333 + AC3gG were prepared by molecular transfer of a PCR amplified fragment encompassing nucleotides 222–2140 of the gG gene into wt333 as previously described [16]. These specific fragments of the gG gene were derived from the mgG deficient AC9 and AC3 variants of wt333, selected by the virus passage in GMK AH1 cells in the presence of the sulfated oligosaccharide muparfostat [16]. The virus variant AC9 was mgG deficient due to the deletion of a single cytosine at position 1655 (GenBank accession number EU018128) resulting in a frameshift and a premature stop codon at nucleotides 1924–1926 while the lack of expression of mgG in AC3 variant was due to insertion of cytosine at position 624 (GenBank accession number EU018100) with premature termination codon at nucleotides 919–921 [16]. The virus recombinant mgG-def + AC9gG (mgG-res), in which the mgG expression was rescued by cotransfection into GMK AH1 cells of the gG gene fragment (nucleotides 222–2140) of wt333 with the mgG-def DNA, was prepared in a similar manner to the mgG deficient recombinant viruses.

### 2.3. Purification of HSV-2 Virions, Glycoprotein gG, and Quantification of Viral DNA

The virus particles were radiolabeled with methyl-^3^H-thymidine (20 µCi/mL) and were purified from infectious extracellular medium and infected GMK AH1 cells by centrifugation through a three-step discontinuous sucrose gradient as described previously [16,32]. Glycoprotein sgG was purified from infectious culture supernatant of infected GMK AH1 cells using the monoclonal antibody 4A5A9 coupled to CNBr-Sepharose beads [23]. Quantification of viral DNA was performed based on real-time PCR analysis as described previously [33].

### 2.4. Virus Replication Kinetics and Forcible Virus Liberation Assays

The GMK AH1 cells growing in 12 well plates were rinsed twice with 1 mL of EMEM and inoculated with HSV-2 at a multiplicity of infection (moi) of 2. Following incubation of cells with the virus for 2 h at 37 °C, the cells were rinsed thrice with 1 mL of EMEM and were incubated for a further 8–24 h in the same medium. The infectious culture medium was harvested and centrifuged at 200× *g* for 5 min. The amount of virus in the supernatant medium was determined by the plaque titration assay [34,35]. The infected cells were gently rinsed thrice with the medium, then scrapped into 1 mL of the medium and subjected to two rapid freeze-thaw cycles at −80 °C ethanol and 37 °C water bath, respectively, to release the cell-associated virus. Following centrifugation at 500× *g* for 5 min, the amount of infectious virus in the supernatant medium was determined by the plaque titration assay. The assay of forced liberation of infectious virus from the surface of infected cells was performed as follows. The GMK AH1 cells were infected with HSV-2 as specified above, and then incubated in EMEM, which at specific times after infection was supplemented with the sulfated oligosaccharide muparfostat, the sulfated polysaccharide heparin, sgG, or with type I-S mucin from bovine submaxillary glands. The area of viral plaques was determined [16] to reflect the cell-to-cell spread of the virus. Briefly, images of at least 20 neighboring viral plaques were captured using a DC300 digital camera (Leica, Heerbrugg, Switzerland) attached to a Diavert microscope with ×2.5 magnification objective (numerical aperture 0.08) (Leitz-Wetzlar, Wetzlar, Germany), and the area of viral plaques was measured using the IM500 software (Leica, Cambridge, UK).

### 2.5. Total Internal Reflection Fluorescence Microscopy

End-on surface immobilization of biotinylated GAGs hyaluronan (b-HA, 23 kDa, INNOVENT e.V., Jena, Germany) and chondroitin sulfate (b-CS; mixture of 70% chondroitin-4-sulfate (CS-A) and 30% chondroitin-6-sulfate (CS-C), 20 kDa, INNOVENT e.V., Jena, Germany) was used to study the binding affinity of wt333 and mgG-def to GAGs. Briefly, supported lipid bilayers containing 1-palmitoyl-2-oleoyl-sn glycero-3-phosphocholine (POPC) and 1% (*w*/*w*) 1,2-dioleoyl-*sn*-glycero-3-phosphoethanolamine-N (cap biotinyl) (DOPE-cap-biotin) were formed from a 0.5 mg/mL liposome suspension on cleaned cover glasses by surface-induced vesicle rupture. The liposomes were produced by the lipid hydration and extrusion method using 30 nm membranes, as described previously [36]. The supported lipid bilayers were incubated with streptavidin (Sigma-Aldrich) for 20 min at a concentration of 25 μg/mL, followed by a 20 min incubation with b-HA (0.2 mg/mL) or b-CS (0.5 mg/mL). The sample was rinsed extensively between each incubation step. In all cases, PBS (137 mM NaCl, 2.7 mM KCl, 8.1 mM Na_2_HPO_4_, 1.5 mM KH_2_PO_4_, 1 mM CaCl_2_ and 0.5 mM MgCl_2_) was used as a buffer.

Preparations of purified wt333 and mgG-def virus particles were fluorescently labeled with PKH26 red fluorescent cell linker dye (Sigma-Aldrich). To this end, the virus suspension was mixed with a 0.5 mM dye-in-ethanol solution in presence of Diluent C (Sigma-Aldrich), which served as a diluting agent (the final virus and dye concentrations were 5.6% (*v*/*v*) and 0.3% (*v*/*v*) respectively). Excess dye was removed by 1-min centrifugation at 2000× *g* through llustra Microspin S-200 HR columns (GE Healthcare, Chicago, IL, USA). Ten microliters of the fluorescently labeled virus particle suspension were added to the GAG-functionalized surfaces (covered with 5 μL of PBS) at approximately 60 min prior to the beginning of the measurements in order to reach equilibrium conditions. One hour time-lapse movies (250 frames, 15-s time interval) were recorded with a Nikon Eclipse Ti-E inverted microscope using a 60× magnification oil immersion objective (numerical aperture = 1.49), an Andor DU879E-CSBV camera, an X-Cite 120 light source (Lumen Dynamics Group Inc., Mississuaga, ON, Canada), and a TRITC Filter Cube (Nikon, Tokyo, Japan). For each surface, time-lapse movies were recorded at 5 different locations on the surface. The time-lapse movies were analyzed with in-house written MATLAB (The MathWorks, Natick, MA, USA) scripts for both Equilibrium Fluctuation Analysis and Single Particle Tracking (SPT) as follows. To extract information on binding kinetics, we used equilibrium fluctuation analysis [37]. Briefly, the software counts the number of newly arrived particles for each frame and measures their individual residence times. It then generates two figures: a cumulative plot of newly bound particles over time, from which we extracted association rates (slope of the linear fit of the association curve), and a histogram of residence times, from which one can obtain information on the dissociation behavior of the particles (as further described in [38]). The obtained association rates were pooled for all 5 recorded positions and divided by the total imaged area and the final viral concentrations of purified viral suspensions in molar according to DNA count (3.7 × 10^10^ DNA copies/mL for wt333 stock solution and 3.8 × 10^9^ DNA copies/mL for mgG-def stock solution). Thus, the slope obtained from the linear fit of the normalized association data can be used to directly compare, qualitatively, the association rate constant (k_on_) of both viruses. For each dataset, association rates of the mgG-def virus mutant were divided by the association rates of the wt333 strain. Averages of the normalized association rates were calculated over 3 datasets and presented with standard deviations.

The recorded time-lapse movies were further analyzed using SPT to quantify particle mobility. Briefly, detected particles between two consecutive frames were linked to construct particle trajectories for which we calculated the mean square displacements (MSDs) values for a range of lag times **Δ**t. The MSD-**Δ**t plots were then fitted using a model for normal diffusion and a model for anomalous diffusion, to extract diffusion coefficients D of the surface bound HSV-2 particles (as further described in [38]). Particles with *D* values higher than a chosen threshold value (10^−4.5^ µm^2^/s for anomalous diffusion and 10^−6.1^ µm^2^/s for normal diffusion) were considered as mobile and served to determine the overall mobile fraction, averaged over 3 datasets and represented with standard deviations.

### 2.6. Binding of Virus to Infected Cells

GMK AH1 cells growing in 12 well plates were rinsed twice with 1 mL of EMEM and inoculated with HSV-2 at moi of 1. Following incubation of cells with the virus for 2 h at 37 °C, the cells were rinsed twice with 1 mL of EMEM and incubated in the same medium for further 22 h. The cells were then gently rinsed with warm EMEM and 1 mL of the same medium comprising viral particles of purified radiolabeled mgG-def or wt333 (both at ~1.7 × 10^7^ of DNA copies) was added to and incubated with cells for 2 h at 37 °C. The cells were then gently rinsed thrice with PBS, solubilized in 0.3 mL of 5% SDS [39], and transferred to scintillation vials for quantification of radioactivity.

### 2.7. Immunogold Electron Microscopy

The HaCaT cells growing in 24 well plates on a Melinex polyester membrane (Agar Scientific Ltd., Stansted, UK) were inoculated with HSV-2 at a moi of 10. After 20 h of infection at 37 °C, the culture medium was removed and cells rinsed once with PBS. The cells were then fixed with 0.25% glutaraldehyde in PBS for 1 h at room temperature. The fixative was removed and the cells were rinsed four times with 1 mL of PBS. Following a 1 h period of blocking of cells with 10% FCS in PBS, 1 mL portions of the same medium comprising 100 µL of monoclonal antibody B11D8 (anti-gB), B1E6 (anti-gE), E5F7 (anti-gC2), or O1C5 (anti-mgG) were added and incubated for 5 h at room temperature under gentle rocking. The cells were then rinsed four times with 1 mL of PBS, and 0.2 mL of PBS supplemented with 10% FCS and 50 µL of the 6 nm gold particle-conjugated F(ab’)2 fragment of goat-anti-mouse IgG (Aurion, Hatfield, PA, USA) was added and incubated overnight at 4 °C under gentle rocking. The cells were then rinsed four times with PBS and fixed with 1 mL of 2.5% glutaraldehyde in PBS for 1 h at room temperature. Following rinsing with 0.05 M Tris-HCl buffer (pH 7.4) supplemented with 2 mM CaCl_2_, the cells were processed for electron microscopy as described by Widehn and Kindblom [40]. The CM10 transmission electron microscope (Philips) was used, and images were captured using the iTEM Image Analysis Platform (Olympus).

### 2.8. Assay to Detect a GAG Lyase Activity

Searching for putative GAG lyase activity in HSV-2 was performed by mixing in PBS the purified radiolabeled GAG chains with purified non-labeled HSV-2 particles. The virus-GAGs mixture was incubated for 20 h at 37 °C, then clarified by centrifugation at 16,000× *g* for 10 min, and the supernatant was centrifuged over filters with 10 kDa cut off. To search for the possible degradation of GAG chains, the radioactivity was quantified in both the pass-through and the filter-retained samples. The GAG chains were also mixed with heat-inactivated (95 °C for 5 min) purified HSV-2 and processed in the same manner to serve as controls. The GAG samples, used in this experiment, were prepared as follows. GMK AH1 cells, seeded the day prior to experiment, were rinsed and incubated for 48 h at 37 °C in sulfate-free EMEM supplemented with 10% normal EMEM, 5% FCS, antibiotics, and 20 µCi/mL of Na_2_^35^SO_3_ (Sulfur-35 radionuclide, specific activity 1050–1600 Ci/mmole, Perkin Elmer). The medium was removed, the cells rinsed twice with EMEM, and the cell-associated GAG chains (proteoglycans) were released from the cell surface by incubation with trypsin (1 mg/mL) in PBS for 15 min at 37 °C. The cell debris was pelleted by centrifugation at 5000× *g* for 5 min, and the supernatant was run through a DEAE-Sephacel column equilibrated with 0.3 M NaCl in 0.2 M phosphate buffer, pH 7.0. Following excessive washing of beads with the same buffer, the retained material was eluted with 2 M NaCl in 0.2 M phosphate buffer. The fractions exhibiting high radioactivity were pooled and incubated at 95 °C for 15 min to inactivate any residual enzymatic activity. The rest of the procedure was carried out as described previously [8,41], except that the treatment step with either heparinase or chondroitinase ABC was omitted to retain both HS and CS chains. The GAG preparation was centrifuged through filters (cut off 10 kDa), and the filter-retained material was solubilized in redistilled water and stored at −80 °C.

### 2.9. Preparation of Cellular Extracts, Electrophoresis, and Immunoblot Assay

Extract of infected GMK AH1 cells was prepared and processed for electrophoresis as described by Finnen et al. [27]. Samples were electrophoresed under reducing conditions on a 4–12% NuPAGE Bis-Tris precast gel (Novex, Carlsbad, CA, USA) and then transferred into PVDF membrane. Detection of viral Us3 and mgG components was performed at a 1:1000 dilution of rat polyclonal anti-Us3 serum [27] and at a 1:500 dilution of monoclonal anti-mgG antibody O1C5 respectively. Peroxidase-conjugated F(ab)_2_ fragments of goat anti-rat or goat anti-mouse IgG (Jackson ImmunoResearch Labs, West Grove, PA, USA) were used at a dilution of 1:1000, and 4-chloro-1-naphtol was used as a substrate.

### 2.10. Statistical Analysis

Multiple unpaired t-test (GraphPad Prism 9.0.2) was used. Experimental data showing the virus titers were transformed into log_10_ values.

## 3. Results

### 3.1. Biological Activities of the mgG Deficient Mutant of HSV-2

**Figure 1:**
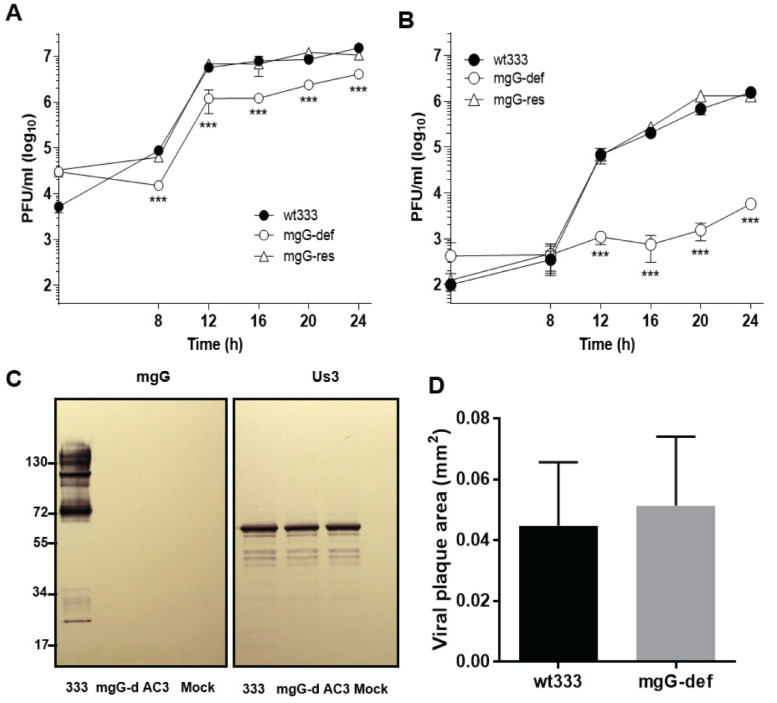
Biological activities of the mgG deficient HSV-2. One-step growth kinetics of the HSV-2 strain 333 (wt333), the mgG deficient recombinant virus (mgG-def), and the mgG-proficient (mgG-res) recombinant virus production in cells (**A**) and in infectious culture supernatant (**B**) of GMK AH1 cells. Values shown are means (±SD) of data from two separate experiments performed in duplicates. Statistically significant differences as related to wt333 at *p* values of <0.005 (***). (**C**) Expression of Us3 protein kinase by the mgG deficient HSV-2. Lysates of GMK AH1 cells infected with wt333 or the mgG deficient HSV-2 mutant viruses mgG-def (mgG-d) or 333 + AC3gG (AC3) were electrophoresed under reducing conditions on a 4–12% NuPAGE Bis-Tris precast gel and electroblotted into PVDF. Viral mgG and Us3 components were detected using monoclonal anti-mgG antibody O1C5 and rat polyclonal anti-Us3 serum respectively. (**D**) Areas of plaques formed by wt333 and the mgG-def mutant virus. The results are shown as the mean size (±SD) of 67 viral plaques captured at 48 h after infection of GMK AH1 cells from six different microscope fields.

We have found previously that the recombinant virus 333 + AC9gG (mgG-def), which is deficient in the mucin-like protein mgG expression due to a frameshift mutation, produced very low amounts of virus in an infectious culture supernatant medium [16]. In the present study, we repeated this experiment but in addition to the mgG-def mutant virus and native wild-type 333 (wt333) strain of HSV-2, we also used a recombinant virus mgG-def + wt333gG (mgG-res) in which the gG expression was rescued by the molecular transfer of the gG gene fragment from wt333 into the DNA of the mgG-def virus mutant. GMK AH1 cells were inoculated with these viruses at a moi of 2 plaque forming units (PFU) per cell, and the yield of infectious virus was assessed in the culture supernatant medium and in infected cells at different time points post inoculation (p.i.). Although the production of mgG-def mutant virus in cells was ~4–6 times lesser than that of wt333 in all time-points after inoculation (Figure 1A), the amount of infectious virus that spontaneously released from cells into the supernatant culture medium was decreased by more than 2 orders of magnitude (Figure 1B). Importantly, the mgG-res, that is, a recombinant virus prepared by the restoration of mgG in the mgG-def mutant virus, produced infectious virus in cells and in culture supernatant in quantities similar to that of wt333 (Figure 1A,B), indicating that the expression of mucin-like protein mgG conferred the efficient release of HSV-2 from cells. Importantly, the low infectious titer of the mgG-def in supernatant medium was not due to the decreased infectivity of viral particles since the specific infectivity values (viral DNA copies/PFU), determined in two preparations of purified virions, were only slightly different, averaging 28 ± 12 for wt333 and 36 ± 21 for mgG-def virus.

Because viral gG and the Us3 protein kinase are expressed from the same bicistronic gene we sought to verify whether frameshift mutations that are present in our mgG deficient virus mutants, would affect the expression of Us3 protein. It has been reported that insertions of relatively large DNA fragments into the gG gene of pseudorabies virus, a related herpesvirus, reduced the expression of Us3 [42], a feature that would complicate the interpretation of data obtained with the mgG deficient viruses. Immunoblot analysis of lysates from infected GMK AH1 cells confirmed that mgG-def and another mgG deficient recombinant virus 333 + AC3gG (AC3) did not express mgG (Figure 1C). This protein is represented in wt333 as the high mannose intermediate (~72 kDa), and the *O*-glycosylated mature forms of gG (mgG) of higher molecular masses. The expression of the Us3 protein was investigated using polyclonal rat anti-Us3 serum that detects a protein of slightly higher molecular mass than predicted for Us3 (~53 kDa) [27]. The mgG deficient mutant viruses produced, like wt333, the Us3 protein (Figure 1C) indicating that the poor release of the mgG-def virions into extracellular medium was not due to the absence or alteration in Us3 expression. Furthermore, we found that the mgG-def virus mutant formed plaques of a size similar to that of wt333 (*p* = 0.08; *n* = 67) (Figure 1D), indicating that the low titer of free viral particles was not due to major defects in replication or the cell-to-cell spread of this virus.

**Figure 2:**
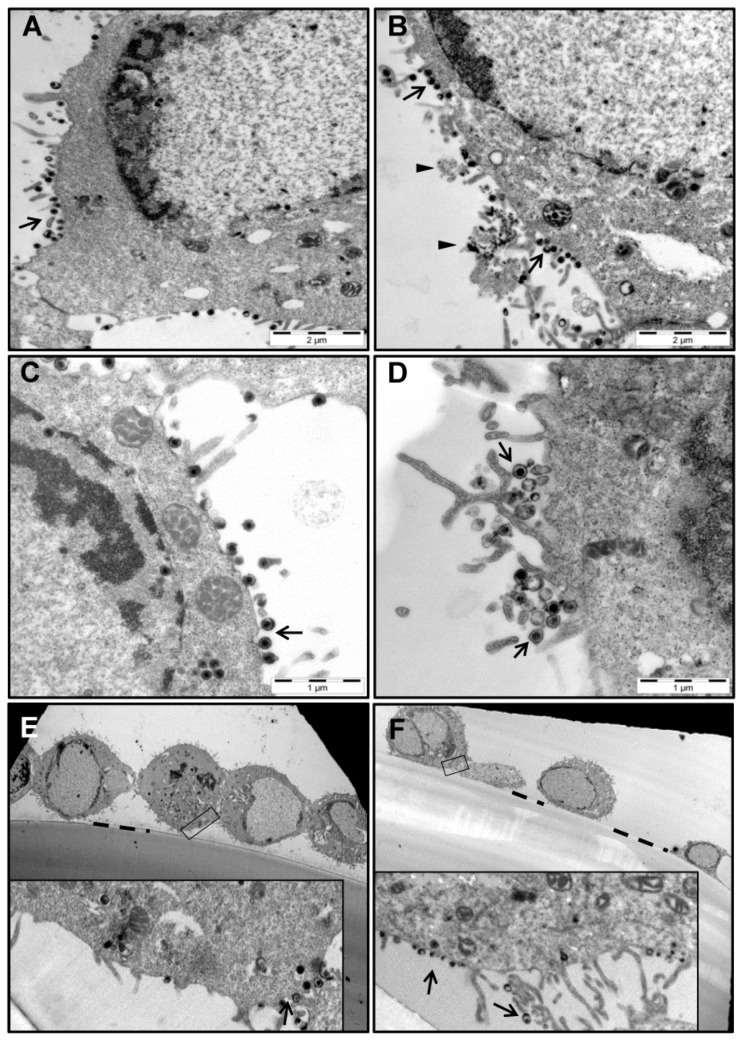
Presence of the mgG deficient HSV-2 virions on the surface of infected cells. GMK AH1 cells were inoculated with the HSV-2 strain wt333 (**A**,**C**,**E**) or the mgG-def mutant virus (**B**,**D**,**F**), and 24 h later processed for electron microscopy. Infected cells shown in images (**A**–**D**) were cut horizontally while these shown in images (**E**,**F**) were cut vertically relative to the Melinex supporting membrane. HSV-2 virions (arrow), extracellular deposits of an electron dense material (arrowhead), and the border between the supporting Melinex membrane and the cell (dashed line) are indicated. The images shown were selected from a pool of over 100 images captured in seven separate experiments.

The results shown in Figure 1 revealed that compared to wt333, the mgG-def mutant virus produced in infected cells approximately 4–6 fold less infectious virus (Figure 1A) which was poorly released into infectious culture medium (Figure 1B). To clarify this issue, GMK AH1 cells infected with the mgG-def mutant virus or wt333, were analyzed by electron microscopy (Figure 2A–F). The cells were propagated on Melinex polyester membranes to be processed for electron microscopy without any scraping or pelleting. We found that the mgG-def virus mutant (Figure 2B,D) produced, like wt333 (Figure 2A,C), mature enveloped virions that were present on the surface of infected cells.

**Figure 3:**
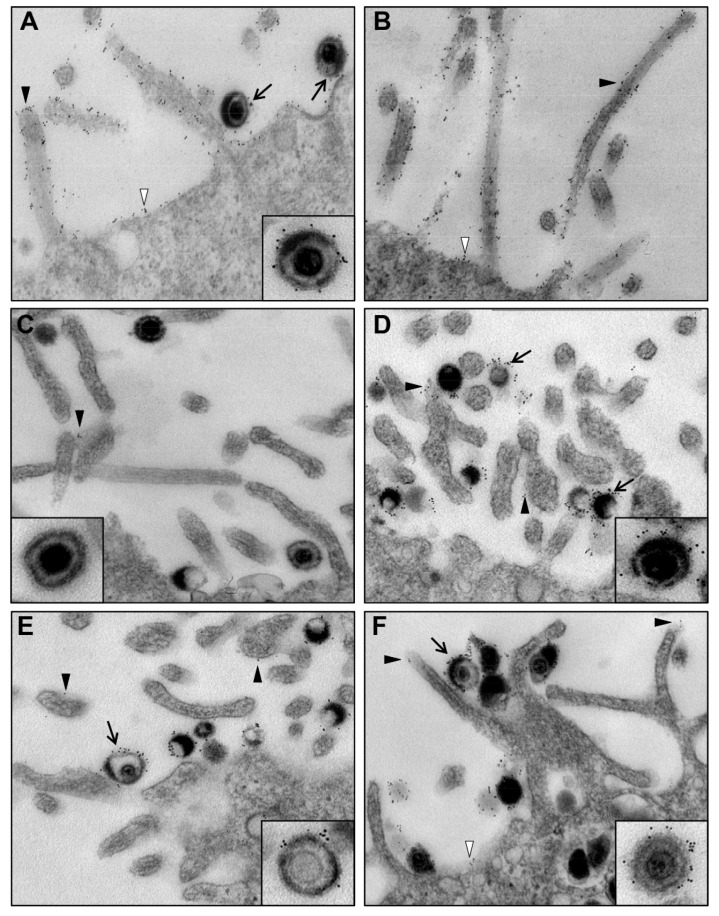
Expression of mgG of HSV-2 at the surface of infected cells. HaCaT cells were inoculated with the HSV-2 strain wt333 (**A**,**B**,**D**,**E**,**F**) or with the mgG-def virus mutant (**C**), and 24 h later the cells were immunogold labeled using monoclonal antibodies specific for mgG (**A**–**C**), gB (**D**), gC (**E**) or gE (**F**) followed by the 6 nm gold particle-conjugated F(ab’)2 fragment of goat-anti-mouse IgG. The HSV-2 virions (arrow), cell plasma membrane (white arrowhead) or cellular protrusions (black arrowhead) decorated with gold particles are indicated. The insets of enlarged virions are derived from separate images.

Since GMK AH1 cells infected with mgG-def produced ~4–6-fold less infectious virus than cells infected with wt333 (Figure 1A), one could expect similar decrease in the mgG-def virion quantity at the cell surface. However, the number of virions per cell, counted on cell surface in images of six infected cells, averaged 58 ± 34 and 66 ± 17 for mgG-def and wt333 respectively suggesting a similar accumulation of mgG-def and wt333 virions. The adherence of cells infected with wt333 and the mgG-def mutant virus to the Melinex support membrane is shown in Figure 2E,F, respectively. To further search for an explanation of the role of mgG in the virus infection of cells, we investigated the expression of this glycoprotein at the surface of infected cells by using an immunogold electron microscopy technique (Figure 3).

In cells infected with wt333, the mgG was expressed on the surface of virus particles and on the cell plasma membrane including long cytoplasmic protrusions (Figure 3A,B). As expected, this specific pattern of mgG labeling with gold particles was absent in cells infected with the mgG-def virus mutant (Figure 3C). The viral glycoproteins gB (Figure 3D), gC (Figure 3E), and gE (Figure 3F) were detected at the surface of viral particles, and the expression of these components at the cell plasma membrane and cytoplasmic protrusions was less abundant than that of mgG. These results indicate that the HSV-2 expresses ample amounts of mgG on the plasma membrane of infected cells and in HSV-2 virions.

### 3.2. Release of the mgG-def Virions from the Surface of Infected Cells

**Figure 4:**
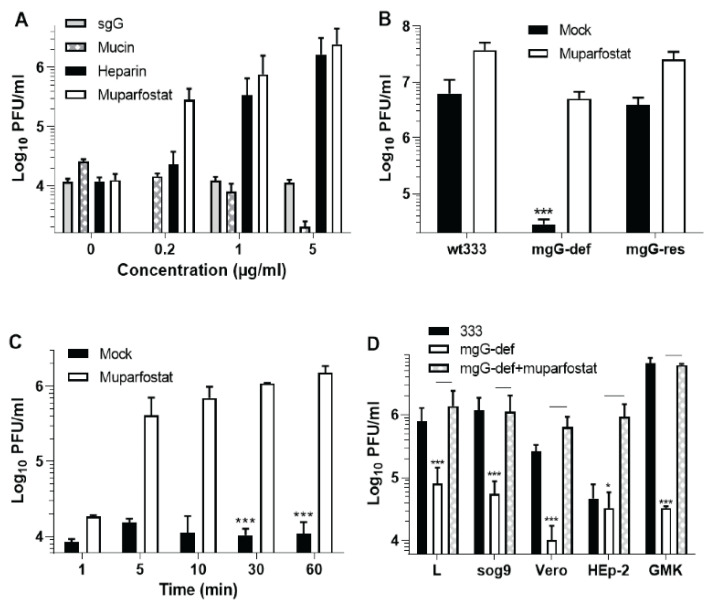
Forcible liberation of the mgG deficient HSV-2 from the surface of infected cells. (**A**) GMK AH1 cells were infected with the mgG-deficient (mgG-def) mutant virus at multiplicity of infection of 1 for 2 h at 37 °C, then rinsed twice with Eagle´s medium and incubated for further 22 h in the same medium supplemented with specific concentrations of secreted gG (sgG) of HSV-2, mucin, heparin, and muparfostat. The concentrations of mucin were 2000 times higher than indicated in the graph. (**B**) GMK AH1 cells were infected with HSV-2 strain 333 (wt333), the mgG-def, and the mgG-proficient recombinant virus (mgG-res) and then incubated with muparfostat (10 µg/mL) as described above. (**C**) GMK AH1 cells were infected with the mgG-def as described in part (**A**) and subjected to short treatments with muparfostat (10 µg/mL) beginning at 24 h after infection. (**D**) Murine L and mutant sog9 cells as well as Vero, HEp-2 and GMK AH1 cells were infected with wt333 or the mgG-def, and the cells infected with the latter virus were subjected to muparfostat (10 µg/mL) treatment as described in part (**A**). The virus titers (PFU/mL) were quantified in infectious culture supernatant medium and all values shown are means (±SD) of data from two to four separate experiments performed in duplicates. Statistically significant differences at *p* values of <0.005 (***) or <0.05 (*).

Knowing that the mgG-def virions are present on the surface of infected cells (Figure 2B), we attempted to forcibly liberate infectious virus by treatment of cells with the following agents: (i) Secreted fragment of gG (sgG) because gG of HSV-2 apart from the virion-associated mgG also comprises sgG that is secreted from infected cells into the medium [19]; (ii) mucin from bovine submaxillary glands because mgG comprises an extensive mucin-like region, and (iii) GAGs or their mimetics such as sulfated oligosaccharide muparfostat or sulfated polysaccharide heparin which are known to compete with cell surface GAG chains of HS and CS for binding sites in HSV attachment proteins [16]. After infection of cells for 2 h at 37 °C, the inoculum was carefully removed and the cells were rinsed and incubated in medium supplemented with these agents for further 22 h, that is, until the development of complete cytopathic effect. Only the GAG-mimicking compounds liberated substantial amounts of infectious virus (Figure 4A). Although the mgG-def virus is known to be relatively resistant to muparfostat and heparin, which was manifested as ~10 fold decreased sensitivity to these compounds as compared to wt333 [16], incubation of cells with muparfostat and heparin (both at 5 µg/mL) substantially enhanced the level of infectious virus in supernatant culture medium (Figure 4A). Remarkably, while the level of wt333 and the mgG-res was enhanced by muparfostat ~6–7 fold, the amount of mgG-def virus in the medium was increased ~200 fold, that is, nearly to the level found with mock-treated wt333 (Figure 4B).

To discriminate whether these enhancing effects of the GAG-mimetic muparfostat were due to direct activation of infectivity of viral particles or facilitation of virus release from the cell surface, the cell-free infectious culture supernatant of the mgG-def virus was mixed with muparfostat (10 µg/mL) and incubated for 1 h at 37 °C. The mean infectious titers found in three experiments were 7.2 × 10^3^ and 6.9 × 10^3^ PFU/mL for the muparfostat and mock treated samples respectively, indicating that muparfostat did not directly stimulate infectivity of the mgG-def virions. Previously we have observed that muparfostat exhibited no permanent inactivation or enhancement of infectivity of wt333. To clarify whether muparfostat could release the mgG-def virus from the cell surface, infected cells (24 h p.i.) were subjected to a short muparfostat treatment. Muparfostat was capable of releasing substantial amounts of mgG-def virus already after 5 min of treatment (Figure 4C). Because such a short duration of treatment may not be sufficient for uptake of muparfostat and stimulation of virion exocytosis, we conclude that the mgG-def virions were present in substantial quantities at the cell surface and that muparfostat forcibly liberated them into the medium.

Liberation of the mgG-def virions by GAG-mimetics suggested that these virions are trapped on the cell surface due to their binding to GAGs, and that the mgG proficient wt333 but not mgG-def virus particles may somehow promote the virus release from cells. To address this issue, we investigated whether wt333 virions express any GAG lyase activity (heparanase, chondroitinase or sulfatase) that could liberate virus particles trapped at the cell surface due to their binding to GAG chains. To this end, purified ^35^SO_3_ labeled GAG chains (~100,000 cpm) were mixed with purified wt333 particles or with heat inactivated (95 °C for 10 min) purified virus particles and incubated for 20 h at 37 °C. The mixtures were then centrifuged over filters with a 10 kDa cut off. Because HS chains expressed in GMK AH1 cells averaged 30 kDa in apparent molecular mass [7], we hypothesized that any GAG lyase activity of HSV-2 may generate low molecular mass cleavage products of GAG chains. By using this approach, we found that incubation of GAGs with both native and heat-inactivated virus particles produced very low levels of filterable ^35^SO_3_ labeled material (<40 cpm) with no clear difference between these two preparations. These data suggest that HSV-2 has no GAG-destroying activity.

To investigate whether the expression of GAG chains would affect release of HSV-2 from infected cells we quantified both spontaneously released and muparfostat liberated virus in the mutant sog9 cells derivative of the GAG proficient murine L cells. Sog9 cells were originally reported to be deficient in expression of HS and CS GAG chains [30], however, these cells were later found to still express CS chains which due to defect in expression of chondroitin-4-*O*-sulfotransferase-1 were ~3 times shorter in chain length and possessed decreased proportion of 4-*O*- and 4,6-*O*-sulfated disaccharide units but increased proportion of 6-*O*-sulfated disaccharides as related to parental cell line [43]. GMK AH1, Vero, and HEp-2 cells were also included in this experiment for comparative purposes (Figure 4D). In comparison with wt333, the defect in release of mgG-def mutant virus from cells was the most pronounced in GMK AH1 cells (~2 log_10_ decrease in infectious titer), and it was also evident in Vero and murine L and sog9 cells (~1–1.5 log_10_). In HEp-2 cells the amount of released mgG-def virus did not differ from that of wt333. Note that the lack of difference between wt333 and the mgG-def in HEp-2 cells was not due to efficient liberation of the mutant virus but rather owing to relatively poor release of wt333 from these cells. Treatment of infected cells (including HEp-2 cells) with muparfostat substantially enhanced the yield of mgG deficient virus in the supernatant medium (Figure 4D). Importantly, the amounts of the mgG-def mutant virus in the supernatant medium of sog9 and L cells were similar. Likewise, treatment of infected cells with muparfostat enhanced the yield of extracellular virus in a similar way, that is, ~20 fold in sog9 and ~17 fold in L cells (Figure 4D). These data indicate that the defect in release of mgG-def virions from cell surface as well as their forcible liberation with GAG mimetic also occurred in sog9 cells which do not express HS but produce CS chains [43].

### 3.3. Interaction of mgG-def Virions with Surface-Bound CS Chains

**Figure 5:**
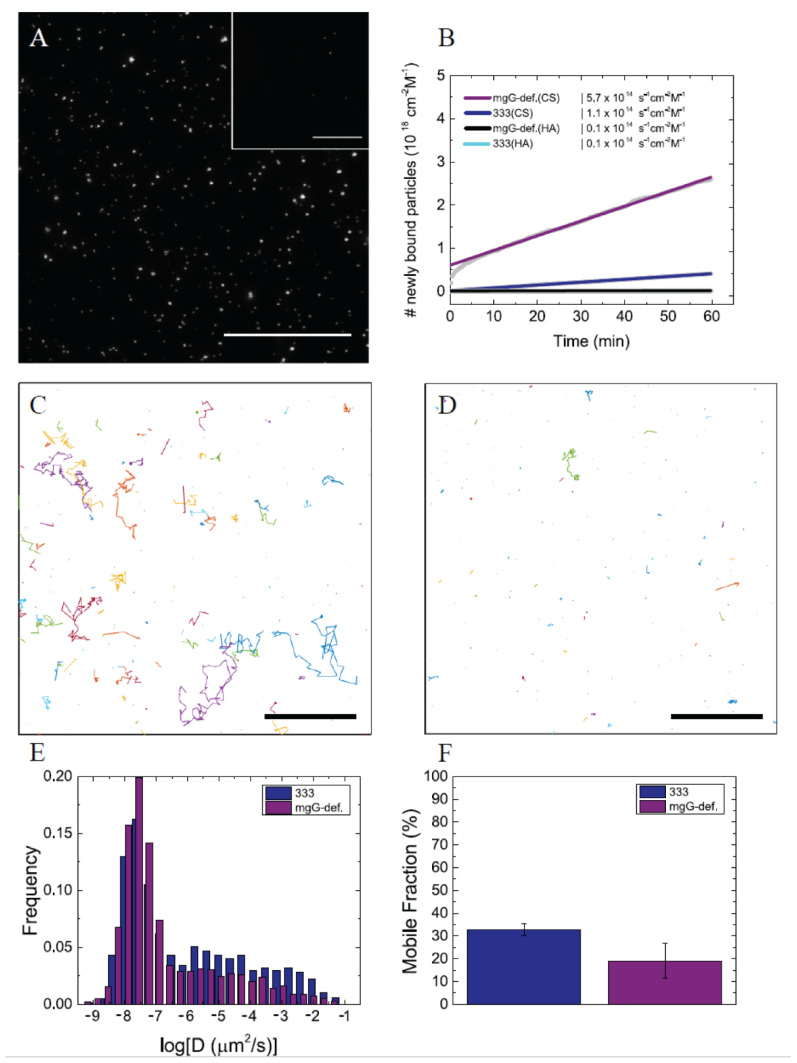
TIRF microscopy to quantify HSV-2 binding and mobility to end-grafted GAG chains. (**A**) TIRF images showing fluorescently labeled HSV-2 strain wt333 particles bound to chondroitin sulfate (CS) and hyaluronan (HA) (inset). Scale bar: 50 µm. (**B**) Cumulative plot of newly bound particles over time, including linear fits, for the wt333 and mgG deficient (mgG-def) mutant virus on CS and HA. The data was normalized to the virus concentration, yielding a slope proportional to the association rate constant (k_on_) and allowing for a direct comparison of the association behavior of both viruses. The association curves were corrected to account for the differences in particle concentration of the two virus suspensions. (**C**,**D**) Mobile trajectories of HSV-2 strain 333 (in **C**) and mgG-def (in **D**) on CS, determined by SPT analysis of a representative TIRF movie. Scale bars: 20 µm. (**E**) Histograms of diffusion coefficients for HSV-2 333 and mgG-def particles diffusing on CS. (**F**) Mobile fractions of wt333 and mgG-def viral particles diffusing on CS as determined from the histograms of diffusion coefficients.

To complement our data shown in Figure 4D with regards to a possible participation of CS chains in interaction with the mgG-def mutant virus, and to further clarify contribution of mgG to the virus-GAG interaction, we used total internal reflection fluorescence microscopy (TIRFM) to study the virus binding kinetics and mobility on surface-bound CS (Figure 5). The GAG chains were end-grafted onto the sensor surface in a brush-like architecture, in order to mimic the attachment of GAGs to proteoglycans. Hyaluronan (HA), a non-sulfated GAG, was used as a negative control. The TIRFM-setup makes it possible to visualize individual surface-bound particles while suppressing the fluorescent background (Figure 5A), allowing for the analysis of virus binding and release events, as well as their mobility, on a single particle level. In this study, the characteristics of the interaction of mgG-def and wt333 with CS were compared.

First, to investigate the association behavior, we measured the rate of arrival of particles to the sensor surfaces. Figure 5B shows association curves of wt333 and the mgG-def mutant virus to CS and HA. On CS, the measured association rate for wt333 was 22.6% ± 3.9% of the association rate of the mgG-def. Both viruses showed very low association to HA (~2.4% of association for mgG-def and ~1.1% for wt333 in comparison to the respective association rates to CS), as expected, for a non-sulfated GAG [38]. For reaction-limited binding kinetics, the measured association rates are directly proportional to the association rate constants $k_{on}$ [38], which allows for a qualitative comparison of the binding propensities of wt333 and the mutant virus. The observed differences in association rates therefore indicate that the mgG-def mutant virus bound to CS approximately 4.5 times more efficiently than wt333. Analysis of the dissociation behavior of the two viruses revealed that once bound only very few virus particles (less than ~3% for both viruses) detached from the CS layer, as observed previously for HSV-1 [38].

In addition to kinetic analysis, we performed single particle tracking (SPT) analysis to quantify virus particle diffusion on the CS surfaces. Visual inspection of the recorded movies revealed that a fraction of CS-bound HSV-2 particles underwent lateral diffusion as illustrated by the individual particle trajectories shown in Figure 5C,D for the wt333 and mgG-def viruses, respectively. Moreover, the wt333 (Figure 5C) appeared to show increased mobility compared to the mgG-def mutant virus (Figure 5D). To quantify this visual observation, we calculated the diffusion coefficients corresponding to the measured trajectories. Data in Figure 5E show the histograms of the calculated diffusion coefficients for both viruses. Both histograms are dominated by a peak at ~10^−8^ µm^2^/s, which we attributed to immobile particles. The non-zero value of this peak is caused by localization errors of the particles [38]. In addition, the histograms show a large tail in the distributions of *D* values with diffusion coefficients ranging up to ~10^−1^ µm^2^/s, which implies that a few particles can travel distances up to 40 µm during the acquisition time of 1 h. The tail distribution of wt333 particles showed higher frequencies at high *D* values compared to mgG-def, which is in line with our initial observation that fast diffusing wt333 particles were more numerous. To illustrate this, we further calculated for each virus the average mobile fraction, determined by the percentage of particles with *D* values higher than a threshold value, which we chose based on the position of the immobile peak in the histograms (see Materials and Methods section for further details). We measured a mobile fraction of 32.8 ± 2.7% for the wt333 strain and 19.1 ± 7.5% for the mgG-def mutant virus (Figure 5F), confirming increased mobility for the wt333 in comparison to the mutant virus. These data demonstrate that the mgG-def virions exhibited much more extensive binding to CS chains compared to wt333 virions, a feature that may limit their lateral diffusion. This suggests that mgG plays a role in balancing the interaction of HSV-2 particles with GAG chains.

**Figure 6:**
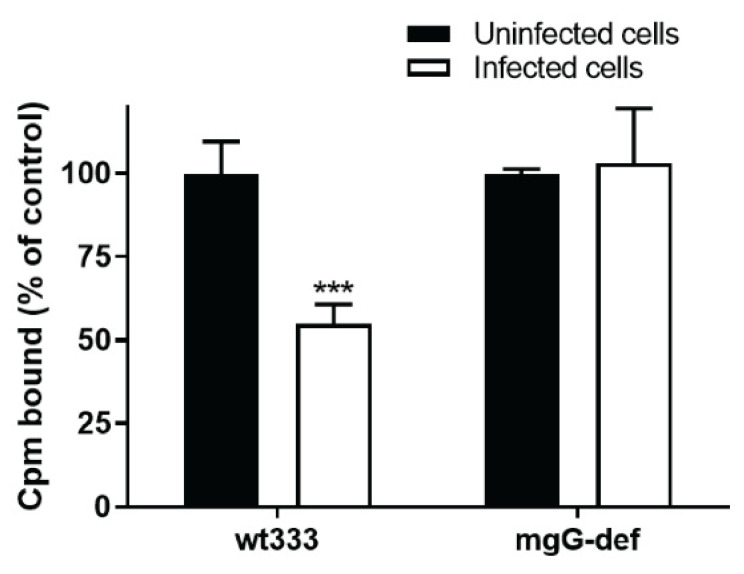
GMK AH1 cells were inoculated with the strain wt333, and 24 h later purified methyl-^3^H-thymidine labeled mgG deficient or wild type virions (adjusted to 1.7 × 10^7^ DNA copies) were added and incubated with cells for 2 h at 37 °C. The results are expressed as a percentage of the number of counts per minute (cpm) bound to infected cells relative to that bound to non-infected control cells. Values shown are means (±SD) of data from two separate experiments performed in duplicates. The mean number of cpm bound to non-infected cells were 1053 and 554 for wt333 and mgG-def virions respectively. Statistically significant differences as related to uninfected cells at *p* values of <0.005 (***).

Furthermore, because the mgG-def mutant virus exhibited extensive GAG-binding activity (Figure 5B), we tested the binding of these virions to infected cells. HSV shuts off the synthesis of most host cell proteins [44] to avoid their interference with the virus life cycle including limitation in the re-binding of newly produced virions to infected cells. GMK AH1 were inoculated with wt333 or were mock inoculated, and 24 h later purified methyl-^3^H-thymidine labeled mgG-def or wt333 virions were added and incubated with cells for 2 h. While wt333 bound more efficiently to non-infected than to infected cells, the mgG-def virions exhibited similar binding efficacy in both kinds of cells (Figure 6). These results suggest that interaction of the mgG-def virus mutant with cells is greatly unbalanced, a defect manifested as efficient virus binding to infected cells.

## 4. Discussion

We report that the mucin-like glycoprotein mgG of HSV-2 balances the virus interaction with GAG chains, a feature required for efficient release of progeny virions from the surface of infected cells. This conclusion is deduced from the following observations. The amount of infectious virus that was spontaneously released from cells into extracellular medium was substantially decreased in the mgG deficient mutant virus as related to native HSV-2, a difference reversed to native HSV-2 level by restoration of the mgG expression in the virus mutant. This difference cannot be attributed to a defect in production of infectious virus since a total yield of the mgG deficient mutant was only ~4–6 times lower than that of native HSV-2. Moreover, our data exclude the possibility that the poor release of mgG deficient HSV-2 from cells was caused by an altered production of viral Us3, a protein expressed from the same bicistronic gene as gG. It is not known whether the coupled expression of Us3 and gG has any biological significance, however, Us3 protein kinase is a known anti-apoptotic component of HSV-2 [45].

How does the mgG of HSV-2 promote release of virus particles from surface of infected cells? Our attempts to detect any GAG-degrading activity associated with HSV-2 particles have failed. However, it has been recently reported that HSV enhanced expression of cellular heparanase, an enzyme that degrades HS chains thus liberating the virus particles trapped at the cell surface [14]. These authors found that the knockdown of heparanase gene in cultured cells decreased the amount of liberated HSV-1 by ~4 fold. Our data indicate that the deficiency in mgG expression reduced the amount of HSV-2 released from GMK AH1 cells by ~200 fold. Furthermore muparfostat (PI-88), a known inhibitor of cellular heparanase [46], did not inhibit the HSV-2 release from cells but instead it substantially enhanced the amount of liberated virus. This suggests that besides an enzymatic degradation of GAG chains, the viral components such as mgG could be needed for efficient release of HSV-2 virions. Indeed, an interaction between the virus attachment components and GAG chains is known to be relatively weak and of electrostatic nature [47,48], and the GAG mimetics such as heparin and muparfostat, which are more extensively sulfated than GAG chains, may easily release the virus particles from their binding to GAGs. Our previous study [48] revealed that interaction of muparfostat with the HSV particles is reversible and non-virucidal and a simple dilution of the virus-muparfostat mixture liberated infectious virus. Furthermore, some intrinsic features of mucin-like proteins may promote the reversibility of this binding. In particular, the mucin-like region forms an extended structure due to the presence of numerous *O*-linked glycans that are frequently terminated with a negatively charged sialic acid residue. Hence, both steric hindrance from this extended domain and electrostatic repulsion between the sialic acid coat and the negatively charged sulfate/carboxylate groups of GAG chains can contribute to the reversibility of the virus binding to GAGs. Since the GAG chains are ubiquitous components of the cell surface, the reversibility of the virus-GAG interaction may counteract trapping of viral particles engaged in non-productive dead-end interactions that prematurely terminates the virus life cycle at the stage of virus attachment to cells or release of progeny virions from the surface of infected cells.

Machiels et al. [49] reported that bovine herpesvirus 4, lacking the mucin-like protein gp180, showed enhanced susceptibility to neutralization by immune sera. Since gC and gB are the GAG-binding components of HSV-2 [32] and the latter protein was reported to mediate the binding of HSV-2 to cells [50], mgG together with gC and gB may form an attachment complex that both promotes and balances the virus interaction with GAGs. Indeed, we have previously found that mgG preserves specific sensitivity of HSV-2 to the GAG-mimicking inhibitors of virus attachment to cells [16]. More importantly, the absence of mgG in virus particles seems to unbalance the virus binding to GAGs, a notion strongly supported by our analysis of interaction between HSV-2 and surface-immobilized CS chains, demonstrating a clearly enhanced association to the GAGs for the mgG deficient virus particles in comparison to the native HSV-2 virus particles. In addition, we demonstrated that CS-bound HSV-2 particles undergo lateral diffusion, a feature that we already reported for HSV-1 [38]. Remarkably, the diffusive activity of the GAG-bound virions appeared to be compromised for mgG deficient mutant virus particles. Although reasons behind this poor mobility of the mgG deficient virions require further clarification it is likely that the lack of the mucin-like mgG protein may facilitate accessibility and/or may enhance capability of the major virus attachment components gB and gC to interact with the GAG layer, a possibility supported by the observed increase in the CS-mgGdef association rates. This effect could in turn lead to an increased number of bonds between the individual virions and the CS chains and thereby compromise the lateral diffusion of the virions. Another possible explanation could be that the mgG glycoprotein itself, through interaction with the GAG chains, plays a role in guiding the virus movement on the cell surface. We believe that the extensive GAG-binding capability that compromised lateral diffusion could also be detrimental for the liberation of the mgG deficient viral particles from the surface of infected cells. This interpretation seems to be in line with our data showing the defect in release of mgG deficient virus particles from sog9 cells which express truncated variants of CS chains as the only type of sulfated GAGs [43]. Moreover, our data showing that the mgG deficient mutant virus, unlike native HSV-2, bound to infected cells as efficiently as to non-infected cells strongly suggest that the viral particles that lack mgG are greatly unbalanced and vulnerable to unfavorable or premature interactions with infected cells. The capability of ionic compounds such as GAG mimetics to liberate this virus suggests that the trapping of the mgG deficient virus is based on its reversible electrostatic interactions with GAGs. However, one cannot exclude that this mutant virus may bind to other negatively charged cellular components of unknown identity.

Besides viral particles, the mgG was abundantly expressed on the plasma membrane of infected cells including cytoplasmic protrusions. This arrangement is similar both to cellular sialomucins which are known to be extensively expressed on microvillus protrusions [51,52] and to the viral mucin-like proteins such as gp2 of equine herpesvirus 1 (EHV-1) [53] and gp150 of murine herpesvirus 4 (MHV-4) [54] that are regarded as the major virus antigens. Abundant expression at the cell plasma membrane of infected cells was also reported for gD of HSV-1 [55], an event related to possible blockage of the viral entry receptors and interference with HSV re-infection [56,57]. It is not known whether extensive expression of mgG on cellular protrusions coincides with the expression of the HSV attachment receptors that is, the GAG-bearing proteoglycans, however, these proteins are known to stabilize the structure of cellular microvillus protrusions (reviewed in [58]). An abundant expression of mgG may protect infected cells against immune response or may help to release an entire infected cell. Transmission of viral infection through infected cells is a known mode of the host-to-host spread of sexually transmitted pathogens [59].

In HSV-1, the mucin-like domain occurs in the amino-terminal portion of the virus attachment protein gC, and our previous data [60] indicate that this region of gC of HSV-1 functions in balancing the virus-GAG interaction and to some extent in promoting the virus release from the surface of infected cells, that is biological activities similar to these of mgG of HSV-2.

It is worth noting that the mgG deficient mutant of HSV-2 shows significant “functional” similarity to variants of other herpesviruses lacking a specific mucin-like protein. In particular, EHV-1 deficient in heavily *O*-glycosylated glycoprotein gp2, exhibited like the mgG deficient HSV-2 an ~250 fold decrease in the amount of infectious virus released into culture medium while a total virus yield was only 5 times less than that of native virus [61]. Furthermore, MHV-4 lacking an extensively *O*-glycosylated glycoprotein gp150 exhibited severe defect in the virus release from the surface of infected cells documented as virion clustering on the cell plasma membrane in form of several layers of tightly packed virus particles [13]. In case of the mgG deficient mutant we did not observe such massive accumulation of virus particles on the cell surface because this virus produced less infectious virus than native HSV-2. One consequence of the impaired release of the mgG deficient HSV-2 (this report), as well as EHV-1 and MHV-4 mutants described above [13,61] is that all these viruses spread mainly through the direct cell-to-cell contact with little dissemination via release and re-adsorption to non-infected cells.

Altogether, our data indicate that the mucin-like envelope protein mgG preserves specificity of the HSV-2 interaction with cell surface GAGs. This activity of mgG seems to include prevention of excessive binding of viral particles to cells and promotion, in a non-enzymatic manner, of its release from infected cells. Our study provides new insights into the biological functions of viral mucin-like proteins that can be exploited in antiviral and vaccine research.

## Data Availability

The data are available from the corresponding author upon request.
